# Terminology Proposal for Adaptive Particle Therapy Classification and Strategies

**DOI:** 10.1016/j.ijpt.2025.101293

**Published:** 2025-12-22

**Authors:** Pieter Populaire, Rita Simoes, Lena Nenoff, Petra Trnkova, Lorenzo Placidi, Kristin Stützer, Eva van Weerd, Francesca Albertini

**Affiliations:** 1 KU Leuven, Laboratory of Experimental Radiotherapy, Leuven, Belgium; 2 UZ Leuven, Department of Radiotherapy, Leuven, Belgium; 3 Proton Beam Therapy Department, University College London Hospitals, London, United Kingdom; 4 OncoRay – National Center for Radiation Research in Oncology, Faculty of Medicine, Dresden, Germany; 5 Helmholtz-Zentrum Dresden-Rossendorf, Institute of Radiooncology – OncoRay, Dresden, Germany; 6 Faculty of Medicine Carl Gustav Carus, TUD Dresden University of Technology, Dresden, Germany; 7 Faculty of Nuclear Sciences and Physical Engineering, Czech Technic l University in Prague, Prague, Czech Republic; 8 Diagnostic Imaging and Oncological Radiotherapy Department, Fondazione Policlinico Universitario A. Gemelli, IRCCS, Rome, Italy; 9 HollandPTC, Delft, The Netherlands; 10 Center for Proton Therapy, PSI, Villigen, Switzerland

## Introduction

Adaptive radiotherapy (ART) is an increasingly prominent concept in modern radiation oncology, offering the ability to adjust treatment in response to patient-specific anatomical and physiological changes. The concept was first introduced in 1995 as a “closed-loop radiation treatment process where the treatment plan can be modified using a systematic feedback of measurements.”^1^ In this manuscript, we use ART to denote a treatment paradigm in which the treatment plan is intentionally modified during the course of radiotherapy in response to observed or anticipated changes in patient geometry, anatomy, or biology, with the aim of maintaining or improving the therapeutic ratio. More recently, ART has gained momentum due to advances in imaging, computing, and automation, which enable such feedback to be implemented more systematically and at different timescales.^2^, ^3^, ^4^, ^5^ Therefore, ART could eventually become a standard of care for some traditionally difficult-to-treat indications. These developments are particularly significant for particle therapy (PT), which is highly sensitive to (anatomical) variations. While PT offers dosimetric advantages over conventional photon therapy, its precision also means that even small deviations from the planned density and/or geometry can translate into clinically relevant changes in water-equivalent path length and thus proton range, with substantial impact on the dose distribution.

Consequently, ART is especially relevant in PT, where maintaining accurate dose delivery demands carefully designed adaptive strategies to ensure adequate target coverage while minimizing dose to organs at risk, given the finite range of particle beams, their associated range uncertainties, and the typically higher computational demands of PT planning.

As ART continues to evolve, a shared and consistent terminology becomes essential for fostering clear communication across institutions and disciplines. Currently, differences in how ART is defined and classified can complicate the comparison of research outcomes, limit the ability to generalize findings, and slow the development of shared best practices. This challenge is further amplified in the context of PT, where diverse adaptation strategies—motivated by clinical, technical, and operational considerations—demand a more precise and flexible classification approach. Efforts to standardize ART terminology are already underway, most notably through the ASTRO White Paper on quality and safety considerations in ART^6^ and the framework proposed by Paganetti et al for proton therapy,^7^ which itself builds on the foundational work of Heukelom and Fuller.^8^ The ASTRO White Paper offers a rigorous and comprehensive framework with a strong emphasis on quality assurance, introducing terminology elements that are broadly applicable across modalities, albeit with a primary focus on photon therapy. Similarly, Paganetti et al combine clinical motivation with technical strategy to propose a structured classification system tailored to proton therapy. These contributions provide an essential foundation for harmonizing ART nomenclature.

Building on this, the present manuscript aims to further unify terminology. Herein, we argue that classification of ART should reflect not only clinical intent but also operational dimensions—namely, the reason for adaptation, the timepoint in the treatment course, and the timescale within which adaptation is performed. These dimensions have direct implications for workflow design and resource allocation**.** For instance, adaptation to account for interfractional setup variation differs considerably in execution, timing, and personnel requirements from adaptation aimed at dose escalation. Conversely, distinct clinical goals—such as organ-at-risk sparing versus target dose intensification—may require similar underlying technical procedures. Some strategies, especially those that do not involve redefining the target volume, may be suitable for implementation by radiation therapists (RTTs) alone, while others may necessitate direct involvement from physicians (MDs) and/or medical physicists (MPEs). Establishing a common terminology that links clinical intent to operational dimensions is therefore a prerequisite for consistent and transparent role definitions, which is one of the key motivations for the framework proposed in this position statement. In addition, we propose a broad terminology which reflects the way treatment plans are technically modified. To facilitate its practical use, we provide illustrative clinical examples that demonstrates the application of the proposed terminology.

Recognizing the relevance of adaptive PT, the European Particle Therapy Network (EPTN) established a dedicated Adaptive PT Task Group at its annual meeting in Manchester in October 2023. This initiative followed a multidisciplinary workshop, during which terminology standardization was identified as a key priority. A terminology subgroup, composed of all co-authors of this manuscript, was formed to lead this effort. This position statement presents the outcome of that work and aims to support communicational clarity and consistency in ART across institutions, disciplines, and research efforts.

## ART classification

**Figure 1:**
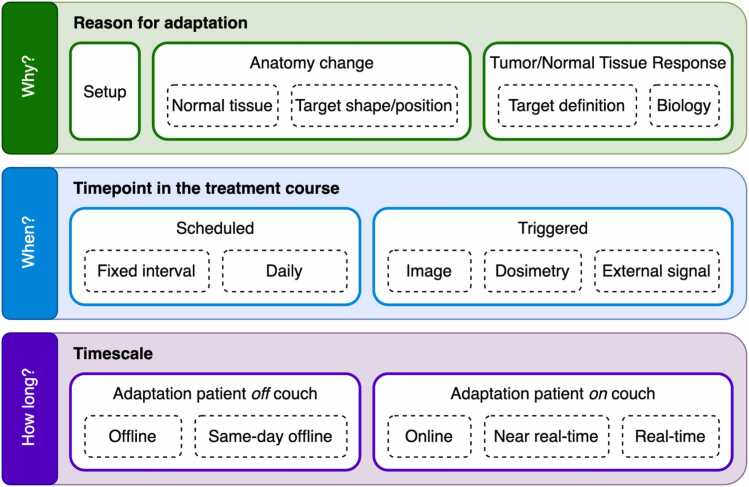
Proposed adaptive radiotherapy classification by the Adaptive Task Group of the European Particle Therapy Network.

We propose 3 different levels that coexist parallel to one another to classify ART, schematically presented in Figure 1.

### Reason for adaptation

**Figure 2:**
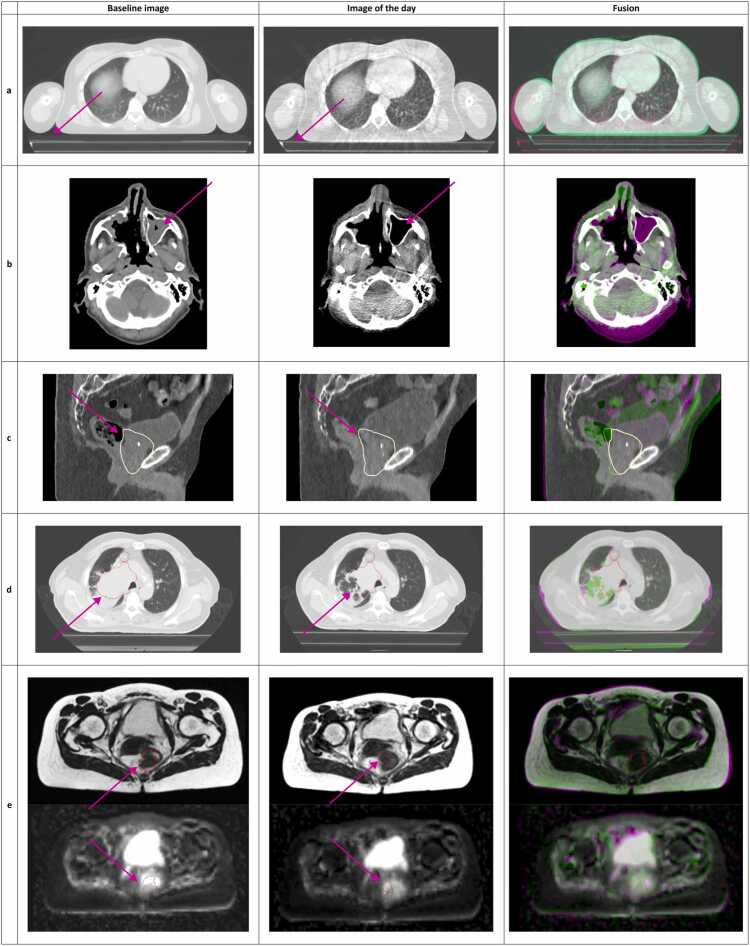
*Reasons for adaptation: example cases.* (a) Changes in setup: the use of a treatment mattrass in the baseline image versus the image of the day without the use of a treatment mattress or vice versa; (b) Changes in anatomy—effect on normal tissues: the baseline image shows filling of the left maxillary sinus, not part of the target volume, which has disappeared at time of image of the day; (c) Changes in anatomy—effect on (normal tissues and) target shape: the different filling of bladder and rectum have shifted the posterior borders of the predefined anatomical borders of the target volume (yellow line, prostate + seminal vesicles) in the image of the day versus the baseline image; (d) Response-based adaptation—redefinition of the target volume: the target volume (red line) was redefined based on tumor shrinkage on the image of the day versus the baseline image; (e) Response-based adaptation—(redefinition of target volume and) biological adaptation: the rectal tumor has shrunken (upper planes) but also shows increase in apparent diffusion coefficient (ADC) values within the gross tumor volume (lower planes) on the image of the day versus the baseline image, these findings could result in adapting the plan for dose escalation on the remaining gross tumor volume.

A first differentiation should be made according to the intent of adaptation: ie, *why* do we want to adapt. We propose 3 categories for intent that are aligned with the operational tasks and possible responsibilities, reflecting the practical workflow and requirements for staff on site and responsibility distribution for ART (Figure 2). As a note, in PT, the below-listed changes are particularly relevant because they modify the water-equivalent path length and thus the proton range.

(1) Changes in setup (Figure 2a): These changes are either patient-related (eg, pain of the patient resulting in inability to sustain the planned treatment position requiring alternate positioning) or device-related (eg, accidental inappropriate settings used on the immobilization device), which result in (expected) changes in the dose distribution. They can be either persistent or occasional (single-fraction) deviations. They are considered a reason for adaptation when they cannot be adequately compensated by standard image-guided couch corrections.
(2) Changes in anatomy: Changes in (local/regional) anatomy can alter the dose distribution. Two effects of anatomical changes are considered, which may occur simultaneously:
  a. Effects on normal tissue (Figure 2b): These refer to modifications in normal tissues. Common examples include changes in weight, occurrence of atelectasis of the lung, pleural effusions, differences in rectal and/or bladder filling and filling of the nasal cavities, as well as the insertion or removal of supportive devices that alter local anatomy and density (eg, esophageal stents or feeding tubes).
  b. Effects on target shape/position (Figure 2c): While, from a clinical point-of-view, the pre-defined target remains the same, its borders may shift due to inter or intra fraction anatomical changes. Examples include reshaping the entire prostate gland based on variations in rectal and bladder filling or redefining the borders of the target volume when treating a pancreatic tumor based on the surrounding structures such as the vessels, bowel, etc.
(3) Response-based:
  a. Redefinition of the target volume (Figure 2d): In response to tumor changes, the target volume may need to be redefined (eg, tumor shrinkage or enlargement). Unlike anatomical changes, this involves a clinical decision to modify the target definition. An example is the shrinkage of a peripheral lung tumor.
  b. Biological adaptations (Figure 2e): Due to (lack of) response of the tumor or even based on observed effects of normal tissues exhibiting toxicity, the treatment plan is adapted to optimize the ratio between tumor control probability and normal tissue complication probability by changing the target prescription and/or clinical goals. An example is tumor dose escalation based on a positron emission tomography performed in the middle of the treatment course.

### Timepoint in treatment course

A second differentiation is to be made at the scheduling and trigger of adaptation, answering the question *when* to adapt. Two major categories are proposed:

(1) Scheduled: There is an adaptation at a *predefined moment* in time or interval, irrespective of findings during the course of the treatment.
  a. Fixed intervals: At a predefined timepoint or interval over the course of the treatment, an adaptive workflow is scheduled. For example, every week a new plan is optimized, or in the middle of the treatment course a single adaptation is performed.
  b. Daily: There is an up-front decision to daily adapt the treatment of the patient, meaning every single treatment day, a workflow for adaptation is executed and delivered. Contrary to longer intervals, the increased frequency warrants a unique operational workflow to achieve this.
(2) Triggered: The adaptive workflow is *triggered* by one of the following. Of note, for the triggered adaptation, a threshold must be established to determine an unacceptable deviation.
  a. Image-based: These triggers are based on a visual evaluation of the anatomy and the subsequent *expected* dose changes, for example, recognition of weight loss, tumor shrinkage, or changes in organ filling.
  b. Dosimetry-based: Unlike image-based triggers, here there is an actual dose-volume *evaluation* which can trigger adaptation. This can encompass both (decreased) target coverage or (increased) dose to the adjoining organs at risk.
  c. External signal based: External signals such as those coming of AI-prediction, range measurements, etc, could also trigger the adaptive workflow.

### Timescale

In this level of classifying ART, we aim to answer the question of *within* which time adaptation needs to occur.

(1) Patient off the couch
  a. Offline: Adaptation is taking more than 1 day. Here, it is possible that the patient is treated with a non-adapted plan even though the adaptive workflow has already been initiated.
  b. Same-day offline: The patient receives a new plan the same day as the adaptive workflow has been initiated, but a patient repositioning between imaging and treatment is performed, that is, the patient does not remain on the couch between imaging and treatment delivery. The frequency with which such adaptations are performed (eg, daily, weekly, or triggered) is described separately by the “timepoint in the treatment course” dimension.
(2) Patient on couch: Adaptation is performed while the patient is on the treatment couch, resulting in minimal workflow disruption.
  a. Online: The adaptation process takes place before initiation of treatment delivery. Note that the daily 3D-image can be acquired both in-room or out of the treatment room as long as the patient stays on the treatment couch.
  b. Near real-time: Adaptation takes place after initiation of treatment delivery but not during beam-on time. This can occur between and within treatment fields.
  c. Real-time: The timescale of adaptation is in sub-seconds to seconds, allowing for adaptation of the treatment plan after initiation of treatment delivery and during continuous beam delivery.

## Adaptive particle therapy strategies

**Figure 3:**
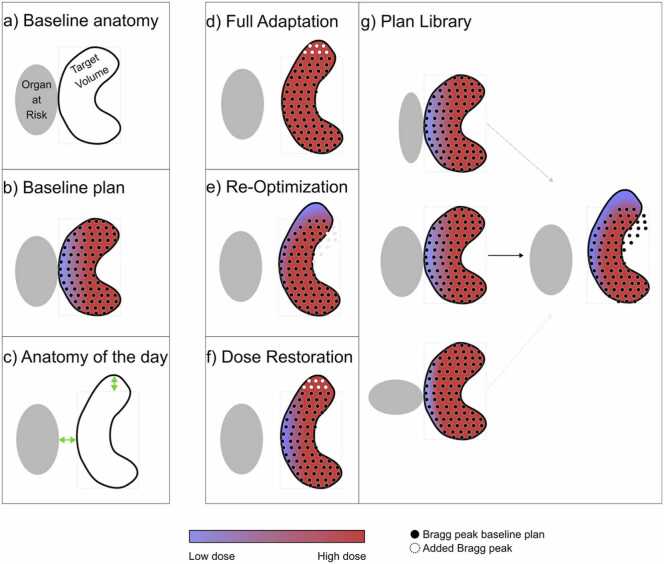
*Sketch of the ART strategies.* (a) The baseline anatomical situation; (b) The baseline plan compromises target coverage due to an overlap with an organ at risk (OAR); (c) In the new daily anatomy, the target has expanded, and there is now a separation between the target and the OAR; (d) Full adaptation involves re-planning the treatment, considering the updated anatomy. This includes generating a new Bragg peak distribution and performing a full optimization. If the anatomy is favorable (as in this example), target coverage can be improved; (e) In the re-optimization approach, specific to the PT plan, the initial Bragg peak distribution is maintained and corrected for changes in daily density. Fluences of partial or complete beamlet distributions can be adjusted. However, since no new Bragg peaks are added, this approach may result in underdosage in areas where the target has expanded, as shown in the upper part of the target; (f) In the dose restoration (dose mimicking) approach, a similar dose distribution to the original plan is achieved for the daily anatomy. This means that target coverage remains compromised in the same way as in the initial plan. However, target coverage in the upper part of the target can be restored, as new Bragg peaks are added; (g) In the plan-library approach, multiple (baseline) CT simulations are performed to account for different anatomical scenarios. The most suitable pre-optimized treatment plan is selected based on the daily imaging. In this example, increased target-OAR separation results in underdosage in the distal part of the target relative to the OAR. *Abbreviations: ART,* adaptive radiation therapy; PT, particle therapy.

Adaptation strategies can be divided in 4 main categories based on different aims, methods, and other considerations: (1) full adaptation, (2) re-optimization (3) dose restoration (dose mimicking), and (4) plan library. A sketch of these categories is reported in Figure 3 below. Among these, re-optimization is developed in the context of PT primary as an efficient way to create the adapted plan.

### Full adaptation

Aim: To meet the clinical goals under the current anatomy, potentially improving plan quality relative to the initial plan.

Method: This approach involves full re-planning, generating a new Bragg peak distribution based on the daily anatomy, and performing a complete plan optimization using the same optimization objective or new ones to better conform to the daily anatomy.

Considerations: The full adapted plan aims to achieve similar or improved plan quality without being constrained to reproduce the original dose distribution. Thus is, it allows leveraging favorable anatomical changes but requires fast dose calculation and optimization algorithms (Figure 3d).^9^, ^10^ While beam angles are typically kept fixed, reporting whether these are adapted is recommended.

### Re-optimization

Aim: To meet the clinical goals using the original Bragg peak configuration, adapting only the beamlet fluences.

Method: The original spot list is retained and may or may not be corrected for daily density changes, with fluence modulation applied to the existing beamlets.

Considerations: The re-optimized plan aims to achieve similar or improved plan quality without being constrained to reproduce the original dose distribution. However, while efficient, this method may fail if the anatomy deviates substantially, in which case additional Bragg peaks may be required to guarantee full target coverage (Figure 3e).^11^, ^12^, ^13^, ^14^, ^15^, ^16^

### Dose restoration (dose mimicking)

Aim: To reproduce the initial dose distribution as closely as possible on the updated anatomy.

Method: A new plan is generated with a dose distribution matching the initially approved plan using updated beam parameters (spot positions, energies, and fluences) based on daily imaging.

Considerations: This approach guarantees to achieve the initial dose and might simplify clinical approval steps. However, it explicitly avoids taking advantage of favorable anatomy as, by design, cannot exceed the quality of the original plan (Figure 3f).^17^, ^18^, ^19^, ^20^, ^21^

### Plan library

Aim: To approximate the original clinical goals by selecting from a pre-optimized set of plans that represent anticipated anatomical variations.

Method: Multiple (baseline) CT scans (eg, with varying bladder filling/cavities fillings) are used to generate a library of plans. On each treatment day, the plan best matching the current anatomy is selected.

Considerations: This approach is widely used in photon radiotherapy and increasingly proposed for PT.^22^, ^23^, ^24^, ^25^, ^26^ However, predefined CTs may not fully capture the daily anatomy, limiting dosimetric accuracy (Figure 3g).

## Examples of defined terminology in clinically delivered ART workflows

### First clinical application of daily online adaptive proton therapy

The first report of clinical implementation of daily online adapted proton therapy was recently published.^9^ Here, for the last 3 to 8 fractions of a patient's treatment, a daily in-room CT was performed, followed by daily contouring by structure propagation, quality assurance, and subsequent full adaptation of the pencil beams. The workflow was performed in regions with rigid anatomy.

Using the previously mentioned terminology and concepts, this is classified as.

• Reason: Changes to setup and anatomy (normal tissue)
• Timepoint: Scheduled (daily)
• Timescale: Online
• Strategy: Full adaptation

### Adaptation protocol for esophageal cancer in proton versus photon radiotherapy trial

In the phase III randomized controlled trial called PROTECT,^27^ patients with esophageal cancer are subject to a weekly repeated 4D CT. Clinically approved contours should be evaluated in the existing treatment plan. In case of underdosage of the target, over dosage of the spinal cord, or hotspots in the body, adaptation is required.^28^ The protocol stipulates a single fraction can be delivered after acquisition of the new 4D CT using the old treatment plan, in case adaptation is required.

Using the previously mentioned terminology and concepts, this is classified as.

• Reason: Changes in anatomy (normal tissue and target)
• Timepoint: Triggered (dosimetry-based)
• Timescale: Offline
• Strategy: Full adaptation

### CBCT-based adaptation in photon-based radiotherapy for prostate cancer

One commonly reported and clinically applied ART workflow is the CBCT-based workflow for patients undergoing photon-based radiotherapy for prostate cancer, such as the Varian Ethos system.^29^ Here, the main reason for adaptation is to tackle the issue of different rectal and bladder filling that can also influence the shape of the clinical target volume.

Using the previously mentioned terminology and concepts, this is classified as.

• Reason: Changes in setup and anatomy (normal tissue and target)
• Timepoint: Scheduled (daily)
• Timescale: Online
• Strategy: Full adaptation

### Stereotactic MR-guided on-table ART (photon-based)

Using MRI-Linac technology allows for daily adaptation with great soft tissue contrast, as utilized in the multicentric phase II NCT03621644 trial.^30^ In this study on pancreatic cancer stereotactic radiotherapy, the ART-workflow was performed in case of dosimetric (OAR constraint violation or insufficient GTV coverage) or anatomical shift between GTV and OAR when adaptation of the treatment plan is expected to improve target coverage. While the protocol aimed for correcting anatomical changes, these unavoidably happened alongside setup changes, which were corrected for in the adaptation as well. This was performed with the patient on the treatment table.

Using the previously mentioned terminology and concepts, this is classified as.

• Reason: Changes in setup and anatomy (normal tissue and target)
• Timepoint: Triggered (image-based OR dosimetry-based)
• Timescale: Online
• Strategy: Full adaptation

### Plan selection based on bladder filling for cervical cancer (photon-based)

As the target volume in external beam radiotherapy for cervical cancer is significantly dependent on bladder filling, a plan library approach has been suggested.^23^ Here, (baseline) images are acquired in (at least) 2 states: full bladder and empty bladder; with corresponding plans generated. Based on daily imaging, the best-fitting, pre-existing plan is chosen for delivery. This approach holds potential to ensure target coverage with reduced PTV margins, while also reducing unnecessary OAR dose.

Using the previously mentioned terminology and concepts, this is classified as.

• Reason: Changes to anatomy (normal tissue and target)
• Timepoint: Scheduled (daily)
• Timescale: Online (selection of plan)
• Strategy: Plan library

## Discussion

In this position statement, we introduced a unified terminology framework for adaptive PT developed by the EPTN Adaptive PT Task Group. As both clinical implementation and research in ART accelerate, the need for a common language—one that enables precise interdisciplinary communication—has become increasingly apparent. Our terminology aims to capture the reason for adaptation, its timing, and the operational complexity of different workflows.

Our proposed classification builds on previous conceptual efforts, including those from Heukelom and Fuller,^8^ the review by Paganetti et al,^7^ and the ASTRO white paper on ART.^6^ While each of these proposed some (level of) terminology, there was a need for a more specific yet comprehensive classification system that also reflects the implications to workflow structure and task allocation within the treatment team. Our framework for categorizing ART addresses this gap by incorporating 3 orthogonal dimensions: reason for adaptation, timepoint in the treatment course, and timescale.

Whereas the timepoint and timescale dimensions (eg, daily versus triggered, offline versus online) apply similarly to PT and photons, the “reason” and “strategy” dimensions exhibit PT-specific nuances. In both photon and particle ART, setup and anatomical changes are common clinical reasons for adaptation, but in PT these same changes often translate into modifications of water-equivalent path length and thus proton range, with a pronounced impact on the distal dose fall-off and neighboring OARs. This enhanced range sensitivity, together with the higher computational cost of PT planning, has historically motivated proton-specific adaptive strategies such as re-optimization, which explicitly control or constrain changes in Bragg peak configuration while still adapting the dose distribution.

In the proposed terminology, we aimed to reflect distinct changes in the operational workflow. For instance, when classified by the timescale dimension: online adaptations demand immediate action and coordinated team involvement, whereas offline adaptations allow for greater scheduling flexibility. Similarly, considering the timepoint in the treatment course, there is a notable operational difference between daily adaptations and those that occur occasionally, whether triggered or predefined. Moreover, by explicitly classifying adaptations according to clinical reason, we provide a framework that may also support differentiated task delegation within the clinical team. For example, adaptations intended to mitigate setup errors often adhere to well-established protocols that may be safely managed by RTTs. Conversely, response-based adaptations involving potential target redefinition or major plan modifications typically necessitate the closer involvement of MDs and MPEs. Intermediate scenarios, such as shape adaptation of target volumes, are increasingly implemented,^31^, ^32^ but currently lack a harmonized legal or professional framework. Additionally, adaptive scenarios where the patient stays on the treatment couch may coincidentally address setup errors alongside other potential reasons for adaptation, further complicating role delegation. In current practice, device-related setup deviations should be corrected directly whenever feasible, with adaptation reserved for residual or non-correctable changes. As rapid and cost-effective ART workflows mature, reliance on manual repositioning may decrease for selected setup-related scenarios.

While this manuscript does not address role delegation in detail, establishing a consensus on this topic remains one of the goals of the EPTN Adaptive Task Group that will be further explored. By formalizing terminology, we aim to facilitate clearer discussions around responsibilities, potentially enabling more consistent and scalable implementation models. In particular, the “reason for adaptation” dimension explicitly separates scenarios that lend themselves to protocol-driven, RTT-led workflows (eg, setup-driven corrections within predefined boundaries) from those that, by their nature, involve clinical decision-making and therefore require direct medical doctor or medical physicist oversight (eg, target redefinition or biological adaptation). As such, the manuscript provides the necessary terminological basis on which future, more formal definitions of roles and responsibilities can be built.

While our proposal for terminology was not developed through a formal consensus methodology, it reflects the multidisciplinary perspectives of the task group and was refined through iterative discussions informed by practical clinical experience. As such, it serves not as a prescriptive model but as a pragmatic and adaptable structure that can be used to standardize communication, support training and protocol development, and foster collaboration within and across institutions. In line with this, we suggest that centers adopt the proposed framework when describing, analyzing, and reporting adaptive PT so that the reason for adaptation, timepoint in the treatment course, and timescale are consistently documented.

We view this work as a first step toward a comprehensive terminology for ART across both particle and photon treatments. As a next step, we plan to extend and refine the framework in collaboration with photon-therapy groups and to seek a formal, Delphi-style consensus process to consolidate definitions and reporting recommendations. We hope that the terminology proposed in this position statement contributes to a more consistent and comprehensive communication and reporting of ART, particularly in the rapidly evolving field of adaptive PT.

## CRediT authorship contribution statement

Populaire Pieter: Conceptualization, Methodology, Investigation, Writing - Original Draft, Writing - Review & Editing, Project administration, Data curation, Visualization. Simoes Rita: Conceptualization, Methodology, Investigation, Writing - Original Draft, Project administration, Data curation. Nenoff Lena: Conceptualization, Investigation, Writing - Review & Editing. Trnkova Petra: Conceptualization, Investigation, Writing - Review & Editing. Placidi Lorenzo: Conceptualization, Investigation, Writing - Review & Editing. Stützer Kristin: Conceptualization, Investigation, Writing - Review & Editing. van Weerd Eva: Conceptualization, Investigation, Writing - Review & Editing. Albertini Francesca: Conceptualization, Investigation, Writing - Original Draft, Writing - Review & Editing, Resources, Supervision.

## Declaration of Competing Interest

The authors declare that they have no known competing financial interests or personal relationships that could have appeared to influence the work reported in this paper.

## Declaration of Generative AI and AI-Assisted Technologies in the Writing Process

During the preparation of this work, the author(s) used ChatGPT as linguistic support and writing aid. After using this tool/service, the author(s) reviewed and edited the content as needed and take(s) full responsibility for the content of the publication.
